# Using a patient portal as a recruitment tool to diversify the pool of participants in COVID-19 vaccine clinical trials

**DOI:** 10.1093/jamiaopen/ooac091

**Published:** 2022-11-08

**Authors:** Tiffany Yuh, Tuhina Srivastava, Danielle Fiore, Harald Schmidt, Ian Frank, David Metzger, Florence Momplaisir

**Affiliations:** Division of Infectious Diseases, Department of Medicine, University of Pennsylvania Perelman School of Medicine, Philadelphia, Pennsylvania, USA; Department of Biostatistics, Epidemiology, and Informatics, University of Pennsylvania Perelman School of Medicine, Philadelphia, Pennsylvania, USA; Department of Psychiatry, University of Pennsylvania Perelman School of Medicine, Philadelphia, Pennsylvania, USA; Department of Medical Ethics and Health Policy, University of Pennsylvania Perelman School of Medicine, Philadelphia, Pennsylvania, USA; Division of Infectious Diseases, Department of Medicine, University of Pennsylvania Perelman School of Medicine, Philadelphia, Pennsylvania, USA; Department of Psychiatry, University of Pennsylvania Perelman School of Medicine, Philadelphia, Pennsylvania, USA; Division of Infectious Diseases, Department of Medicine, University of Pennsylvania Perelman School of Medicine, Philadelphia, Pennsylvania, USA

**Keywords:** diversity, minority, COVID-19, vaccine, recruitment, patient portal

## Abstract

The coronavirus disease 2019 (COVID-19) pandemic has disproportionately affected racial/ethnic minorities in the United States, who are underrepresented in clinical trials. We assessed the feasibility of using the University of Pennsylvania Health System electronic health record patient portal to diversify the pool of participants in COVID-19 vaccine clinical trials. The patient portal was used to send invitations to eligible individuals living in zip codes with high rates of racial/ethnic minorities. The 5614 invited consisted of 96.7% black, 1.3% Hispanic/Latinx, and 1.5% white. The overall response rate was 5.4%, with lower response rates among Black (3.8%) and Hispanic/Latinx (9.6%) as compared to white individuals (91.6%). Among respondents, black individuals had lower rates of interest in participating (26.7%), as compared to white (65.8%) and Hispanic/Latinx (71.4%) individuals. Of 115 respondents who expressed interest, 9 enrolled in the clinical trial, which included 6 black, 3 white, and 1 Hispanic/Latinx. During phone outreach to nonresponders and decliners, common reasons for declining included mistrust of the COVID-19 vaccine, underlying health conditions, and logistical barriers to trial participation. Because of low rates of patient portal account activation and use, compounded with vaccine hesitancy, this method yielded a small number of interested individuals.

## BACKGROUND AND SIGNIFICANCE

The coronavirus disease 2019 (COVID-19) pandemic has disproportionately affected racial and ethnic minority groups in the US including Black, Latinx, and indigenous people, who have experienced higher rates of SARS-CoV-2 infections and higher mortality rates.^1^ With the rapid spread of COVID-19, there was a push to develop vaccines quickly, and pharmaceutical companies had to rapidly recruit participants for clinical trials. Given that minority communities bore much of the burden of the pandemic, the US Food and Drug Administration urged enrollment of racial and ethnic minorities in the vaccine trials.^2^

Historically, racial and ethnic minorities have been underrepresented in clinical trials.^3^^,^^4^ However, clinical research that does not include participants with diverse backgrounds can lead to results that are not generalizable across the entire population. In addition, individuals may feel a sense of distrust towards research findings if the study was not performed in their racial/ethnic group. Having a diverse pool of participants can help increase acceptance of the vaccine among communities where vaccine hesitancy is high.^5^

Several studies have evaluated the use of an electronic health record (EHR) patient portal message for recruiting participants into clinical research, given that one can easily query the EHR to identify patients that meet inclusion criteria.^6^^,^^7^ It is also a low-cost, quick, and secure platform to engage with patients. We assessed the feasibility of using a patient portal to recruit racial/ethnic minorities into COVID-19 vaccine clinical trials.

## METHODS

### Study design, setting, and participants

This is a mixed-methods study including a descriptive analysis of responses to electronic patient portal messages for clinical trial recruitment, and a thematic analysis of qualitative patient interviews to examine reasons for declining to be involved in the clinical trial. This study took place through the University of Pennsylvania Health System (UPHS), a multihospital academic health system in Philadelphia and the surrounding region. UPHS uses the EHR system Epic (Epic Systems Corporation) and its electronic patient portal MyChart, through which patients and providers can communicate. In June 2020, the EHR was used to identify individuals who were ages 40 and older, living in zip codes with high rates of SARS-CoV-2 test positivity and high rates of racial/ethnic minorities living within an accessible distance from the clinical trial study office, with a risk factor associated with severe COVID-19 disease, and an active MyChart account. The included risk factors for severe COVID-19 disease were obesity, diabetes, heart disease, heart failure, kidney disease, and lung disease. Approval for this study was obtained from the University of Pennsylvania Institutional Review Board.

### Protocol

Eligible individuals were sent a MyChart message inviting them to participate in the Moderna COVID-19 vaccine trial. Individuals could respond by indicating whether they were interested or declined to participate. The message was sent an additional 2 times to participants who did not respond to the message. A member of the study team made follow-up phone calls to nonresponders to invite them to participate in the clinical trial and gather their reasons for or against enrollment. Follow-up phone calls were also made to those who declined in order to gather their reasons for declining.

### Analysis

Descriptive data on race and ethnicity were collected at each step of the recruitment process. Qualitative data on individuals’ reasons for or against enrollment were coded by 2 study team members using NVivo (QSR International). The kappa was 0.69, indicating good inter-rater agreement.

## RESULTS

Overall, of the 237 333 UPHS patients residing in Philadelphia, 166 427 (70.1%) have activated a patient portal account. When breaking this down by race and ethnicity, 63.2% of black patients, 81.1% of white patients, and 65.3% of Hispanic/Latinx patients have an activated patient portal account.

Through EHR identification, 13 779 patients fit the inclusion criteria of age 40 and older, living in selected zip codes with high rates of SARS-CoV-2 test positivity, with a risk factor associated with severe COVID-19 disease. Of these, 5614 (40.7%) individuals had activated MyChart accounts and were sent an electronic message inviting them to participate in the vaccine trial. Of these individuals, 5426 (96.7%) were black, 83 (1.5%) were white, 3 (0.1%) were Asian, 94 (1.7%) were multiracial, 8 (0.1%) identified as other, and 73 (1.3%) identified as Hispanic/Latinx (see Table 1).

**Table 1.** Patient portal response rates by race/ethnicity

	Black	White	Hispanic/Latinx	Total
	(*N* = 5426)	(*N* = 83)	(*N* = 73)	(*N* = 5614)
No response	5220 (96.2)	7 (8.4)	66 (90.4)	5313 (94.6)
Declined	151 (2.8)	26 (31.3)	2 (2.7)	186 (3.3)
Interested	55 (1.0)	50 (60.2)	5 (6.8)	115 (2.0)
Enrolled	6 (0.1)	3 (3.6)	1 (1.4)	9 (0.2)

*Note*: Data are expressed as *N* (%).

Only 301 (5.4%) of messaged individuals responded. When comparing rates of response across different racial/ethnic groups, 96.2% of black and 90.4% of Hispanic/Latinx individuals did not respond as compared to 8.4% of white individuals. Among respondents, 55 of 206 (26.7%) blacks, 5 of 7 (71.4%) Hispanic/Latinx, and 50 of 76 (65.8%) whites expressed interest in participating in the clinical trial. Among the total 115 respondents who expressed interest, 24 agreed to a screening visit, and 9 were ultimately enrolled in the trial, which consisted of 6 black, 3 white, and 1 Hispanic/Latinx individual.

Phone calls were made to 277 nonresponders and 110 decliners. A variety of reasons for declining were cited, with the most commonly discussed being COVID-19-specific vaccine mistrust (37.3% of coded interview content), which included concerns about the speed of vaccine development, the experimental nature of the vaccine, efficacy, ingredients, side effects, and the lack of sufficient information about the vaccine. Other commonly raised reasons for declining were concerns about underlying health conditions (31.4%) and barriers to clinical trial participation (13.4%), which included a lack of time, the need for social distancing during the COVID-19 pandemic, a fear of needles, other obligations, lack of transportation, and lack of compensation. Additional reasons for declining included not wanting to be experimented upon within a research study (8.5%), mistrust of the government and leadership (4.5%), general mistrust of vaccines (3.8%), wanting to speak with their physician first (2.8%), and older age (2.1%).

## DISCUSSION

Recruitment of racial and ethnic minorities into clinical trials can be challenging, particularly during a pandemic that calls for a swift rollout. Although patient portal messages are a rapid and inexpensive way to target large numbers of racial/ethnic minorities, we found that minority groups disproportionately lacked access to the portal. A study done in an academic primary care practice in Chicago showed similar lower rates of patient portal activation among racial/ethnic minority patients.^8^ Within those with activated accounts, we found that Black and Hispanic/Latinx individuals had much lower rates of response to the message as compared to white individuals, suggesting decreased patient portal use. Prior studies have also shown decreased patient portal usage among minority groups and vulnerable populations.^9^^,^^10^

Several studies have been published evaluating the use of a patient portal to recruit minority patients. One study focused on recruiting primary care patients in the Detroit area for a trial testing the effectiveness of a colorectal cancer screening decision support program and found that in comparison to whites, black individuals had a lower odds of opening the portal message (odds ratio [OR = 0.46]), clicking the link in the message (OR = 0.75), and agreeing to participate in the trial (OR = 0.85).^11^ Another study based at UT Southwestern used a patient portal to recruit participants to join a Research Recruitment Registry and found that black individuals (OR = 0.73) and Hispanic/Latinx individuals (OR = 0.67) were less likely to agree to participate than white individuals.^12^ It is difficult to make direct comparisons between our study and others given the different inclusion criteria and methods, but while our data suggest similar patterns, it found even more substantial differences in engagement between racial/ethnic minorities and whites. The particularly low rates of engagement within minority groups in our study may be because we targeted our recruitment at a specific subset of the population, living in zip codes in Philadelphia with high rates of SARS-CoV-2 test positivity and high rates of racial/ethnic minorities. People living in these neighborhoods are likely more heavily exposed to negative social determinants of health that impact their engagement, such as housing instability, limited transportation, decreased technological literacy, etc. Strategies to lower the digital divide in electronic patient portal use are needed. Possible methods could include educating providers and staff to offer patient portal access to all patients, creating promotional materials about patient portal benefits, having a tablet available for immediate patient portal account activation in the clinic or hospital setting, and developing training interventions around how to use the patient portal.

In addition, in follow-up phone calls to patient portal message nonresponders and decliners, patients declined involvement in the clinical trial due to mistrust of COVID-19 vaccines, the health care system, medical research, and the government. This is consistent with prior studies on the underrepresentation of minorities within research and COVID-19 vaccine hesitancy among minority groups.^2^^,^^13–16^ A patient portal message may not adequately address the fears that minority patients have. Researchers should address the barriers to clinical trial participation that vulnerable groups disproportionately experience, such as through providing reimbursements for childcare and travel, facilitating transportation, or creating mobile units that meet the subjects where they are. Patient portal messages should be translated into languages other than English and could include additional information that addresses fears and logistical barriers, as well as describes the benefits of participation. Having the portal message originate from the patient’s trusted medical provider may also help in addressing issues of mistrust.

This study was limited in that among the nonresponders, we are unable to tell how many patients did not open the portal message. Therefore, it is difficult to deduce whether recruitment challenges may be primarily attributed to low patient portal use, or whether patients read the message and did not respond due to other barriers such as vaccine hesitancy or fears regarding research participation. Nonetheless, black and Hispanic/Latinx individuals had a significantly lower rate of response than white individuals, suggesting that outreach via a patient portal message was not an optimized method of recruitment into COVID-19 vaccine trials.

## CONCLUSION

Because of low rates of patient portal account activation and use, compounded with vaccine hesitancy, recruitment of racial/ethnic minorities into COVID-19 vaccine clinical trials through patient portal messaging yielded a small number of eligible individuals. Strategies to increase the enrollment of racially/ethnically diverse groups in vaccine clinical trials could include efforts to increase the activation of and use of the patient portal in minority groups, optimization of the patient portal messaging, and identification of alternative methods of community outreach. This must be combined with approaches to address concerns of trial participation and vaccine hesitancy.

## Funding

The Penn Office of the Provost grant number RRP-7821186469.

## AUTHOR CONTRIBUTIONS

FM, HS, IF, and DM conceived and planned the study. DF cleaned and assembled the data. TY and TS coded the interview transcripts. TY analyzed the data and wrote the paper with input from all authors.

## CONFLICT OF INTEREST STATEMENT

None declared.

## Data Availability

The data underlying this article are available in Dryad Digital Repository at https://doi.org/10.5061/dryad.vhhmgqnxp.
